# Pseudomembranous Trigonitis: A Common but Underrecognized Urological Entity

**DOI:** 10.1155/2010/269254

**Published:** 2010-12-01

**Authors:** M. Stavropoulos, A. G. Papatsoris, C. Konstantinidis, M. Chrisofos

**Affiliations:** 2nd Department of Urology, School of Medicine, University of Athens, Sismanoglio Hospital, Sismanogliou 1, 15126 Athens, Greece

## Abstract

Pseudomembranous trigonitis is the term used to describe squamous metaplastic changes of the bladder trigone, which affect nearly 40% of adult females. We present the characteristics of this underrecognized clinical entity and encourage further relevant research.

## 1. Introduction

Pseudomembranous trigonitis is the term used to describe squamous metaplastic changes of the bladder trigone [1]. Such changes are present in up to 40% of adult females (<5% in men) [1]. It was first described in 1905 by Heymann as “cystitis trigoni” and since then several terms have been used, reflecting continuing uncertainty upon this common entity [2]. Herein, we highlight the characteristics of pseudomembranous trigonitis according to the relevant literature with the objective of increasing clinical suspicion.

## 2. Etiopathogenesis

Squamous metaplastic changes of the trigone are observed usually in adult women of childbearing age [3]. These squamous changes of the trigone are nonkeratinizing, in contrast with the keratinizing squamous changes of leukoplakia, which are detected elsewhere in the bladder and require regular followup cystoscopy (as a potential precursor of squamous cell carcinoma) [3, 4].

It has been demonstrated that pseudomembranous trigonitis in women develops under hormonal impact while the metaplastic urothelium undergoes cyclical menstrual changes similar to the vaginal epithelium. Pacchioni et al. [5] performed estrogen and progesterone receptor assays in fresh frozen and paraffin embedded bladder biopsies. Estrogen receptors were identified only in the trigone in relevance with squamous metaplasia. A clear correlation between the presence of steroid receptors at the squamous metaplasia of the trigone was demonstrated. Interestingly, men receiving estrogen therapy develop squamous metaplasia of the penile urethra. Russell et al. [6] studied 21 transsexual men who received oestrogens prior to gender change surgery. Histological examination of the removed anterior penile urethra revealed squamous metaplasia in 15 cases (71%). It seems that the trigone has an embryological derivation distinct from the remainder of the bladder, allowing the trigonal urothelium to respond to estrogenic stimulation [7]. 

Bacteria cystitis has been associated with the development of pseudomembranous trigonitis, especially in cases of recurrent urinary tract infections [1, 8]. There seems to be a link between pseudomembranous trigonitis and recurrent urinary tract infections (UTIs). The fact that patients suffering from recurrent UTIs benefit by oestrogen therapy may be linked to the hormonal aspect of pseudomembranous trigonitis. Recently, the Cochrane Database of Systematic Reviews published a meta-analysis which demonstrated that vaginal oestrogens reduced the number of UTIs in postmenopausal women [9]. Moreover, Long and Shepherd [10] revealed that vaginal metaplasia of the trigone was present in the majority (72%) of bladders examined at autopsy from adult women passing away from nonurological diseases. Histological evidence of chronic inflammation was demonstrated significantly more frequently in bladders showing vaginal metaplasia. HPV genital infection has failed to correlate with pseudomembranous trigonitis. Allen et al. [11] examined 18 females with HPV infection and concomitant pseudomembranous trigonitis and found HPV positive bladder biopsy in only 2 cases.

## 3. Diagnosis

The diagnosis of pseudomembranous trigonitis is done by the cystoscopic image of a white patch of tissue in the trigone [1, 7]. This endoscopic feature is feasibly recognized in the urological practice. Histological examination confirms the diagnosis by revealing layers of stratified squamous epithelium, in contrast with the layers of the normal trigonal urothelium, which include the basal, intermediate, and superficial layer [1]. Towards the surface of pseudomembranous trigonitis, cells become progressively elongated, their nuclei increasingly smaller, and their content of cell organelles reduced. The squamous surface cells, linked by desmosomes, retain many longitudinally arranged fine filaments together with an occasional degenerate nucleus. The mitotic index of pseudomembranous trigonitis is significantly higher than normal (0.17% versus 0.%, resp.) [1]. The histological findings of pseudomembranous trigonitis may also be present in cases of cystitis cystica [12]. Regarding the use of imaging techniques, ultrasonography may reveal a thickening of the bladder neck [13].

Pseudomembranous trigonitis may cause the frequent symptoms of urinary frequency and urgency symptoms. In a prospective study, Murakami et al. [14] took and examined patch biopsies of 44 women complaining of dysuria and frequency. Bacteriuria was present in almost all patients with mild or moderate squamous metaplasia. The study revealed various degrees of squamous metaplasia as well as submucosal fibrosis. These histological changes were more prominent in patients with worse symptoms. Hence, both symptomatic and asymptomatic patients with pseudomembranous trigonitis share similar endoscopic findings; however, these are more prominent in the presence of symptoms. It would be interesting to grade the endoscopic and/or biopsy findings in pseudomembranous trigonitis and correlate them with the presence and severity of lower urinary tract symptoms.

There seems to be an association between urgency/frequency symptoms and/or pelvic pain syndrome symptoms and pseudomembranous trigonitis. A prospective study evaluated female patients presenting with a long history of urinary frequency and chronic urethral and/or pelvic pain [15]. A total of 103 women with a median age of 46 years (range 21 to 84) were included in the study and all patients had pseudomembranous trigonitis findings at cystoscopy.

## 4. Treatment

Several antibiotic regimens have been used to relieve lower urinary tract symptoms in patients with pseudomembranous trigonitis. Recently, Burkhard et al. [15] studied the efficacy of doxycycline in 103 such patients. They received 100 mg doxycycline twice daily for 2 weeks, followed by 100 mg once daily for another 2 weeks. In 30% of the cases complete response was recorded while 41% of the patients reported improvement of the symptoms. In 8 of the 31 patients that consented to followup cystoscopy pseudomembranous trigonitis resolved completely while in 12 cases a decrease in the degree of squamous metaplasia was revealed. 

Recently, endoscopic treatment with the use of laser fibers has been introduced. In a randomized prospective study [16], 62 women with pseudomembranous trigonitis, confirmed on biopsy, underwent treatment with either end-firing (Group 1) or side-firing (Group 2) Nd:YAG laser (energy setting: 30 W). Results were significantly better for the women of the first group (*P* < .001). Followup cystoscopy and biopsies in symptom-free patients did not reveal squamous metaplasia.

We are currently conducting a randomized prospective comparative study between *per os* clarithromycin (500 mg per day) and intravesical cystistat (40 mg of sodium hyaluronate per week) in women with pseudomembranous trigonitis. Sodium hyaluronate is a derivative of hyaluronic acid that replaces the deficient glycosaminoglycan (GAG) layer of the bladder wall. It is the traditional agent for GAG substitution therapy based on existing theories about urothelial dysfunction [17]. Sodium hyaluronate has been safely administered with success for the treatment of chemical and radiation cystitis as well as interstitial cystitis [18, 19]. In our study patients are assessed with a validated symptoms score questionnaire as well as with biopsies of the bladder trigone. Relevant preliminary and followup results are warranted.

## 5. Epilogue

Although pseudomembranous trigonitis affects a large proportion of female population, few patients seek medical consultation and many are asymptomatic. Still, the true meaning and cause of pseudomembranous trigonitis remain elusive. The absence of an obvious causative factor makes treatment difficult while the recent use of end-firing Nd:YAG laser has demonstrated promising results. 

Interestingly, the relevant literature upon pseudomembranous trigonitis is extremely scant. Therefore, studies are needed in order to understand better this very common but underrecognized entity and provide adequate treatment.
